# Monthly Summary

**Published:** 1873-08

**Authors:** 


### MONTHLY SUMMARY.

---

*On the use of Artificial Respiration and Transfusion as a means of Preserving Life.*—Dr. T. Lauder Brunton, of St. Bartholomew's Hospital, presents in an interesting and valuable paper, a review of various important physiological experiments and observations, and show how they may be utilized in the treatment of disease. Quoting Huxley, he says, " Life has but two legs to stand upon, the lung and the heart; for death through the brain is always the effect of the secondary action of the injury to that organ upon the lungs or the heart." The experiments of the Abbe Fontana, Legallois, and Brown-Sequard have shown that headless trunks, isolated portions of the body, or the head alone, may be kept alive, or even restored to life, by a proper supply of oxygenated blood; and not only have nerves and muscles been made to retain vitality, but livers have secreted bile and lung excreted carbonic acid hours after they were excised from the body.

A point of great practical importance is that the parts do not all die at the same time, and, as the brain and spinal cord generally die first, the heart may pulsate regularly after all respiratory movements have ceased and consciousness and reflex action are entirely lost. If, under such circumstances, respiration be kept up artificially, the heart continues beating, and the circulation of arterial blood through the brain may gradually restore its power, respiration recommence, and life be securely re-established. Schiff's experiments have demonstrated that animals may be kept alive, after almost entire destruction of the medulla, or after the injection of water under high pressure into the cranial cavity. Hence it is evident that we may hope for the best results from the use of artificial respiration in some of those cases of apoplexy where an extravasion almost instantly arrests the respiratory movements, either directly, by destroying a part of the medulla, or indirectly, by causing compression of the brain.

In poisoning by wooara, hydrocyanic acid, strychnia, etc., as well as by the bites of snakes, artificial respiration is invaluable as the only means of affording time for the excretion of the toxic agent. Where the poison exists in large quantities, or is excreted very slowly, requiring hours or even days before it can be got rid of, the obvious plan of treatment would be to remove the poison along with the blood in which it is circulating, instead of waiting for its slow removal by the emunctories; and here transfusion comes to the aid of artificial respiration.

---

*A New Method for Healing Ulcers*—(Dr. Nussbaum, *Wien. Med. Presse*, May 4, 1873).—The author claims to have sucessfully treated upwards of sixty cases of chronic, extensive and otherwise intractable leg-ulcers, by the following simple procedure. It is at least worthy of a trial. The patient is first narcotized, and then around the ulcer of the leg or foot, a finger's breadth from its margin, an incision extending down to the fasciæ is made; numerous blood-vessels are divided, and a severe hemorrhage ensues unless a fine pledget of lint be packed into the cut and the entire ulcer strongly compressed. The packing with lint is also necessary to prevent union of the cut edges by the following day. Upon the second day the bandage and lint are removed from them; until a cure is affected a simple water-dressing is applied.

The author states that an astonishing change can be seen, even in the first twenty-four hours; the ulcer, which yesterday threw off quarts of thin, offensive, ichorous pus, furnishes to-day not more than a tablespoonful of thick, non-offensive, healthy pus. The old ulcer becomes rapidly smaller, healing from the margin towards the centre, and is healed in a short time, but the cut is changed into a broad circular sore, which also speedily cicatrizes.

The great diminution of the secretion, and other favorable changes occurring in the ulcer, find an explanation from the fact that the circumcision has divided dozens of large, abnormally widened blood-vessels. Time is thus given for the lessened nutritive material, which previously was carried off by the excessive secretion, to be transformed into cells and connective tissue; in other words, granulations are formed, which fill up and heal the deep ulcer.

Without claiming this as a radical method, the author assures us that the cure is much more rapid, and the cicatrix becomes more elastic and resisting than in the ordinary means applied, which usually require so much time that the patients depart with half-cured ulcers, soon to find themselves in their previous deplorable condition.—*Med. Times.*

---

*Neuralgia and Sciatica Cured by Turpentine—Turpentine Pearls.*—Doctor Martinet, in a memoir presented by him to the Faculty of Medicine, affirms that he has cured fifty-eight cases of neuralgia and sciatica out of seventy, by the use of the essence of turpentine. The great efficacy of this agent is not to be doubted in the above named affections; and what is remarkable, the benefit is almost always felt from the first doses. But this medicine has such a disagreeable odor, such a harsh and pungent taste, that it is quite impossible to take it pure. Dr. Clertan has contrived to enclose it in very thin and transparent gelatine, in the form of little globules, of the size of peas, to which he has given the name of turpentine pearls. Dr. Clertan's "turpentine pearls" are as readily swallowed in a little water as pills. This agreeable form has made the essence of turpentine quite popular; and now there is not a physician (so says the announcement) in France who does not resort to turpentine pearls in the treatment of neuralgia and sciatica. Trosseau always prescribes turpentine in this form.—*Richmond and Louisville Med. Jour.*

---

*The Physiology of The Nervous System.*—The review of "Fournié on the Nervous System," in the last number of *The Journal of Psychological Medicine*, brings out the following arguments: 1st. The possibility of establishing the physiology of the nervous system upon a natural basis. 2d. To determine the nature and functions of the nervous system. 3d. The number and classification of the intrinsic functions and the functions that compose the nervous system. These subjoined facts are noted by Fournié: That the function of the cerebral powers of relation, the cerebral function of reproduction and nutrition, represent all the possible transformation produced on the brain in functional movements. That the brain furnishes a power that controls organic life. This he calls "incitation motrice."

This "incitation motrice" is the functional product of the brain, and is also given: 1st. To the apparatus of relation. 2d. To the organ of nutrition. 3d. To the organ of reproduction. But at the same time that it gives its produced function the brain does not cease to be a living special organ; that is to say, an organ perceiving and recollecting.

---

*Fatal Poisoning from Carbolic Acid.*—A woman in Louisville, Ky., had a mammary tumor excised. Four days afterwards her nurse gave her a tablespoonfull of carbolic acid, diluted with an ounce of water. She complained immediately of its burning, and the nurse tried to provoke vomiting, without success. In less than ten minutes she was unconscious, pupils contracted and insensible, respiration 50, pulse 150, clammy perspiration. Death in two hours.—*Am. Practitioner.*

